# Supplementary material for: The COVID-19 Pandemic Impact on the Psychophysical Health of Post-Menopausal Women: A Cross-Sectional Study
Source: Medicina (Kaunas). 2023 Jun 15;59(6):1154. doi: 10.3390/medicina59061154 (PMC10304877; doi:10.3390/medicina59061154)
Supplement: Supplementary file 1 [file medicina-59-01154-s001.zip › medicina-2370416-supplementary.pdf]

SUPPLEMENTARY MATERIALS

MP-nCoV19 questionnaire (English version)

QUESTIONNAIRE MP-nCoV19

1. Data of birth: __/__/____

2. Where do you live? ________________

3. Where have you lived during COVID-19? _________________

4. What is your highest educational qualification?
   - Primary school
   - Secondary school
   - High school
   - Bachelor degree
   - PhD

5. What is your marital status?
   - single
   - married
   - never married
   - divorced
   - widow

6. What is your current employment situation?
   - retired
   - stable job
   - occasional job
   - state allowance
   - unemployed

7. Do you have children? If yes, how many?
   - Yes: _________________
   - No

8. What is your weight now (in kg)? ______

9. What was your weight before COVID-19 (in kg)? ______

10. WHat is your height in cm? __________

11. Do you have any medical condition? (you can choose multiple options)
    - hypertension
    - diabetes
    - immunitary system disorders (e.g. HIV)_________________________
    - oncological disease (which one?)________________________________
    - other conditions (which one?)
      ____

12. How much have you been worried about not having access to healthcare due to COVID-19?

    Answer referring to a range from 0 to 4, with 0 lower worry, 4 higher worry

0	1	2	3	4

13. Have you ever felt anxious during COVID-19?

    Answer referring to a range from 0 to 4, with 0 lower anxiousness, 4 higher anxiousness

0	1	2	3	4

14. Have you ever been in hospital or in quarantine due to COVID-19?
    - Yes (write down if hospital/quarantine…): _______________
    - No

15. How much have you been worried about your own or your family's health due to COVID-19?

Answer referring to a range from 0 to 4, with 0 lower worry, 4 higher worry

0	1	2	3	4

16. How much have you worked during COVID-19?
    - Same as before
    - More than before
    - I worked differently than before (e.g. Smart working…)
    - Less than before
    - I could not work

17. How often are you engaged in physical activity?
    - Everyday
    - 1-3 times/week
    - Rarely

18. How often have you been engaged in physical activity during COVID-19?
    - Everyday
    - 1-3 times/week
    - Rarely

19. How often do you usually drink alcohol?
    - Everyday
    - 1-2 times/week
    - 1-2 times/month
    - Never

20. How often have you drank alcohol during COVID-19?
    - Everyday
    - 1-2 times/week
    - 1-2 times/month
    - Never

21. Do you smoke?
    - >10 cigarettes/day
    - <10 cigarettes/day
    - I quit

- Never smoked

22. If yes, how much have you smoked during COVID-19?
    - >10 cigarettes/day
    - <10 cigarettes/day
    - I quit

23. Have your food habits changed during COVID-19?
    - Yes
    - No

24. If yes, how?
    - I started eating without schedule
    - I started eating more food
    - I started eating less food
    - I started eating unhealthier food
    - I started eating healthier food
    - I started ordering takeaways more often
    - I started cooking more often

25. How much do you usually sleep?
    - <6 hours
    - 6-8 hours
    - >8 hours

26. How much have you slept during COVID-19 every night?
    - <6 hours
    - 6-8 hours
    - >8 hours

27. If you have a partner, how do you rate the quality of your romantic relationship?
    Answer referring to a range from 0 to 4, with 0 lower quality, 4 higher quality

0	1	2	3	4

28. If you have a partner, how do you rate the quality of your romantic relationship during COVID-19?
    Answer referring to a range from 0 to 4, with 0 lower quality, 4 higher quality

0	1	2	3	4

29. When was your last period? Write down the year/month __________

30. Are you taking any hormonal or non hormonal menopausal therapy? If yes, specify the name and dosage:
    - Yes: ____
    - No

31. If you are taking hormonal replacement therapy, from how long?
    - <1 year
    - 1-5 years
    - >5 years

32. Do you take any other medication?
    - Yes (Write down name and dosage):
      ____
    - No

33. How often do you usually suffer from menopausal symptoms (hot flashes, vaginal dryness, mood disorders…)?

    Answer referring to a range from 0 to 4, with 0 lower frequency, 4 higher frequency

0	1	2	3	4

34. How has been the intensity of your menopausal symptoms (hot flashes, vaginal dryness, mood disorders…) during COVID-19?

    Answer referring to a range from 0 to 4, with 0 lower intensity, 4 higher intensity

0	1	2	3	4

35. Have you ever felt the need for emergency gynecology visit during COVID-19?
    - Yes
    - No

36. If yes, did you manage to reach a gynecologist during COVID-19?
    - No
    - I had a telephone assessment
    - in ER

- yes, by a gynecologist in the hospital
- yes, by a private gynecologist
